# Supplementary material for: Biosynthesis of GMGT lipids by a radical SAM enzyme associated with anaerobic archaea and oxygen-deficient environments
Source: Nat Commun. 2024 Jun 19;15:5256. doi: 10.1038/s41467-024-49650-x (PMC11186832; doi:10.1038/s41467-024-49650-x)
Supplement: Supplementary file 1 — Supplementary Information file [file 41467_2024_49650_MOESM1_ESM.pdf]

Supplementary information

Biosynthesis of GMGT lipids by a radical SAM enzyme associated with anaerobic archaea and oxygen-deficient environments

Yanan Li[1,2], Ting Yu[3], Xi Feng[1], Bo Zhao[1], Huahui Chen[1], Huan Yang[4], Xing Chen[5], Xiao-Hua Zhang[5], Hayden R. Anderson[6], Noah Z. Burns[6], Fuxing Zeng[3*], Lizhi Tao[2*], Zhirui Zeng[1*]

[1]Department of Ocean Science and Engineering, Southern University of Science and Technology, Shenzhen 518055, China

[2]Department of Chemistry, Southern University of Science and Technology, Shenzhen 518055, China

[3]Department of Systems Biology and Institute for Biological Electron Microscopy, Southern University of Science and Technology, Shenzhen 518055, China

[4]State Key Laboratory of Biogeology and Environmental Geology, China University of Geosciences, Wuhan 430078, China

[5]Frontiers Science Center for Deep Ocean Multispheres and Earth System, College of Marine Life Sciences, Ocean University of China, Qingdao 266003, China

[6]Department of Chemistry, Stanford University, Stanford 94305, USA

***Corresponding authors**: Fuxing Zeng (zengfx@sustech.edu.cn), Lizhi Tao (taolz@sustech.edu.cn), and Zhirui Zeng (zengzr@sustech.edu.cn)

Supplementary Figures 1-12
Supplementary Table 1-4
Supplementary References

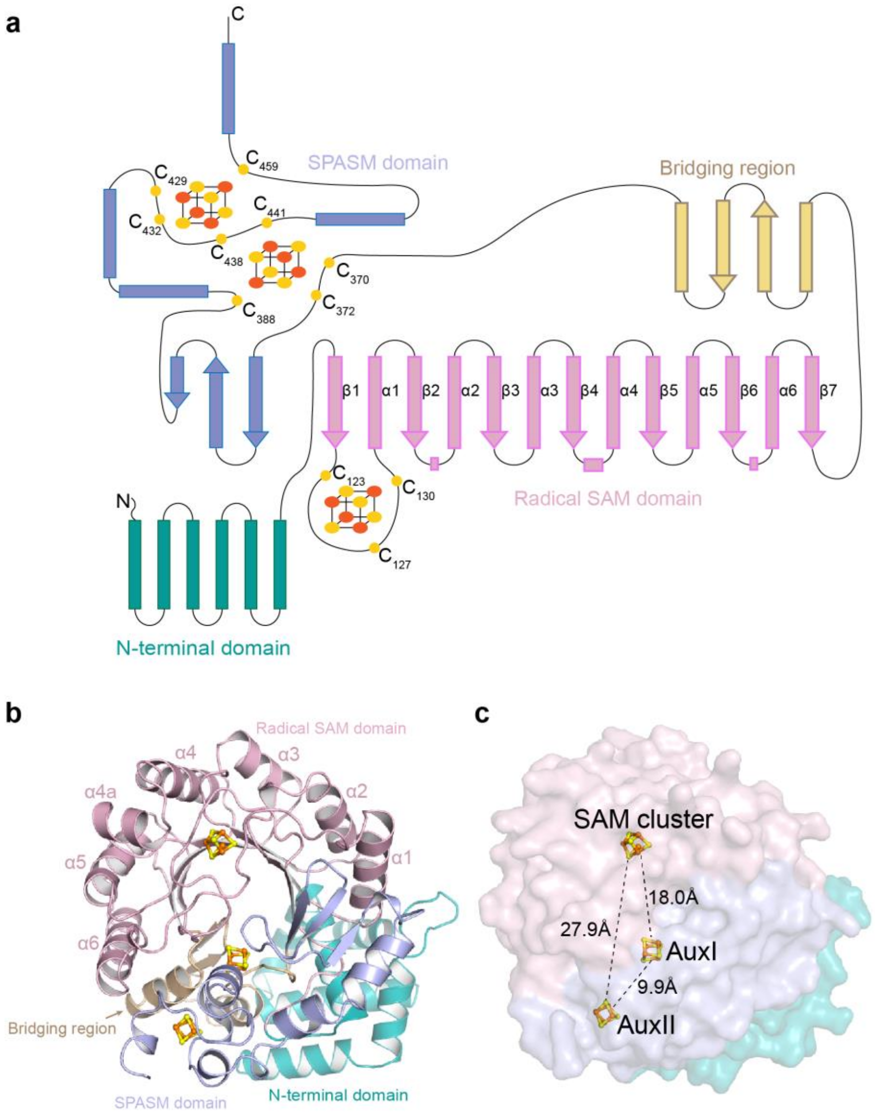

Supplementary Fig. 1 | Distribution of [4Fe–4S] clusters in Gms predicted model. (**a**) Topology diagram of Gms (gene ID: METOK_RS04425), the orange dots represent iron ions while the yellow dots indicate sulfide ions. (**b**) The radical SAM domain (lightpink) forms a partial TIM barrel that is laterally closed by the auxiliary cluster-containing SPASM domain (lightblue). The bridging region and N-terminal domains are shown in wheat and teal, respectively. (**c**) Placement of the three [4Fe–4S] clusters, shown in ball and stick representation (Fe, orange; S, yellow). Distances are calculated between the nearest atoms.

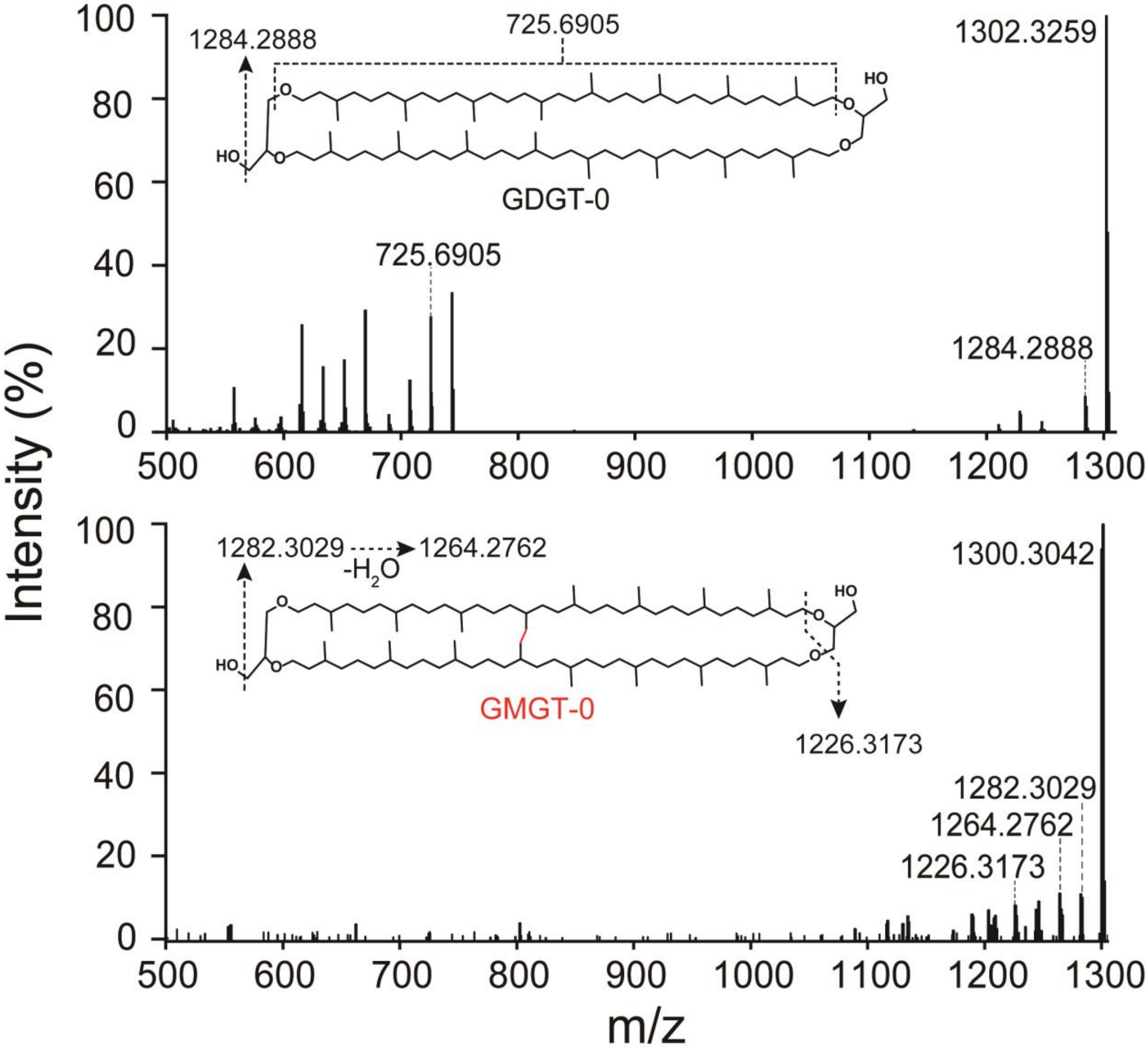

Supplementary Fig. 2 | Mass spectra of GDGT-0 and GMGT-0 isomers in the *in vivo* assays. MS-MS showing the fragmentation patterns of GDGT-0 and GMGT-0.

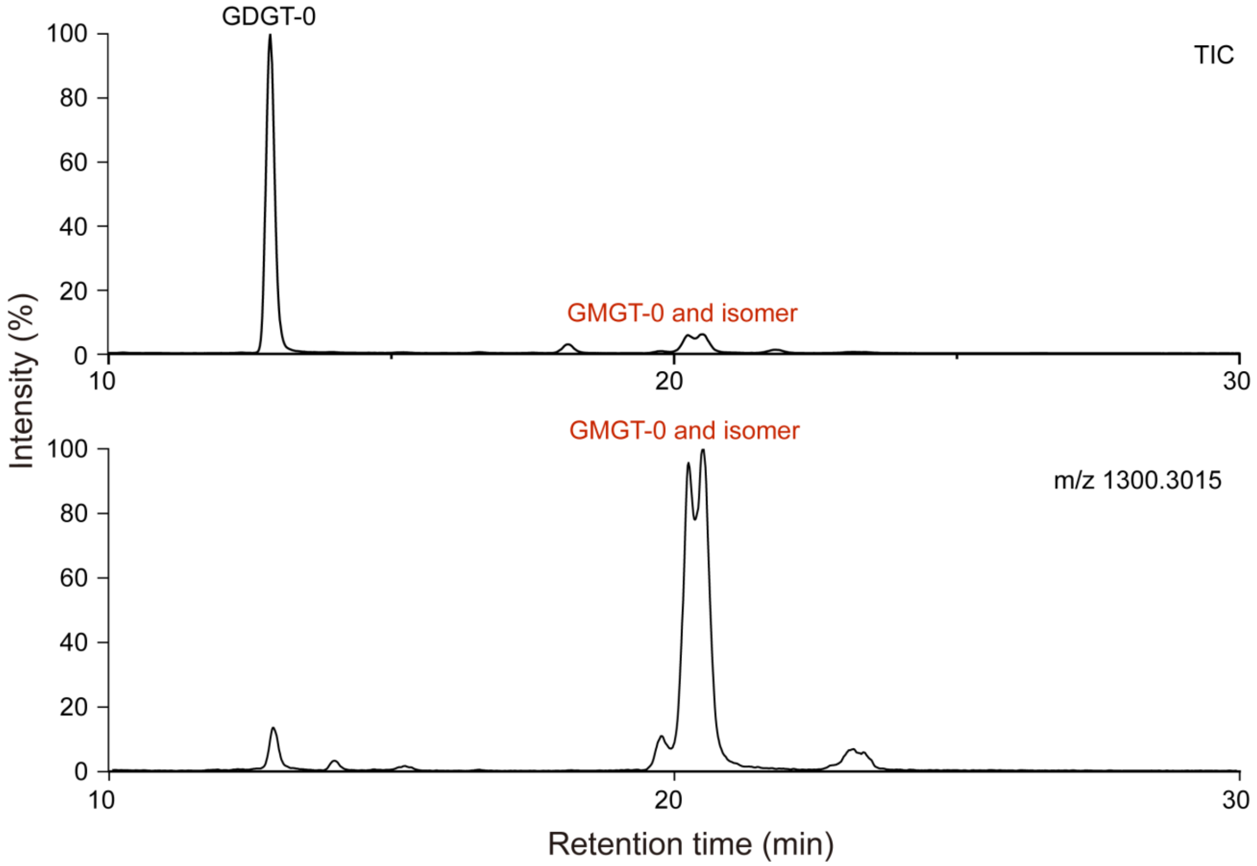

Supplementary Fig. 3 | Gms synthesized GMGT-0 isomer in the *in vitro* assay. LC-MS extracted ion chromatograms of the *in vitro* activity assay. The above figure is the total ion chromatogram (EIC) of 10-30 min, and the below figure is the chromatogram of *m/z* 1300.3015.

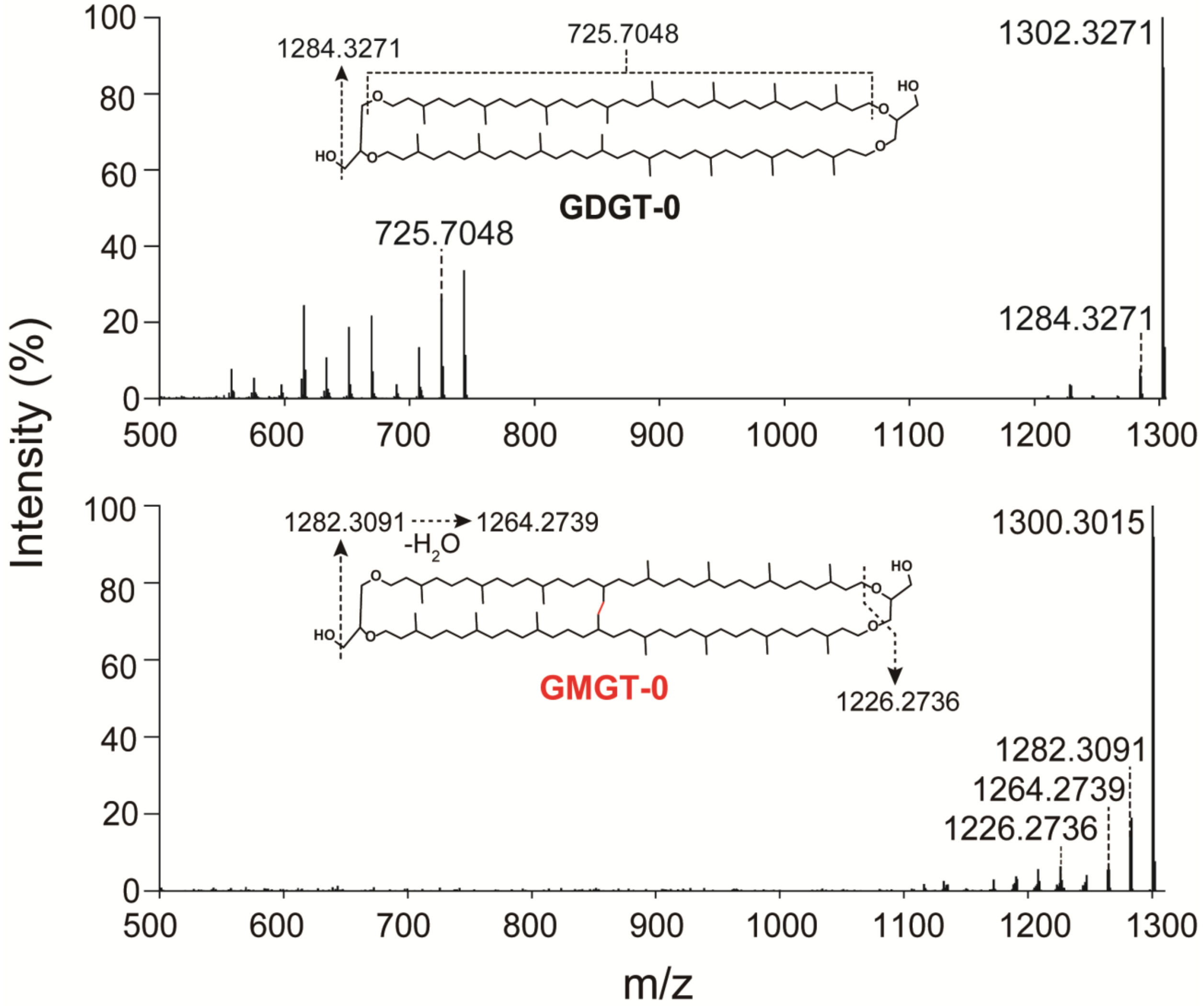

Supplementary Fig. 4 | Mass spectra of GDGT-0 and GMGT-0 isomers in the *in vitro* assays. MS-MS showing the fragmentation patterns of GDGT-0 and GMGT-0.

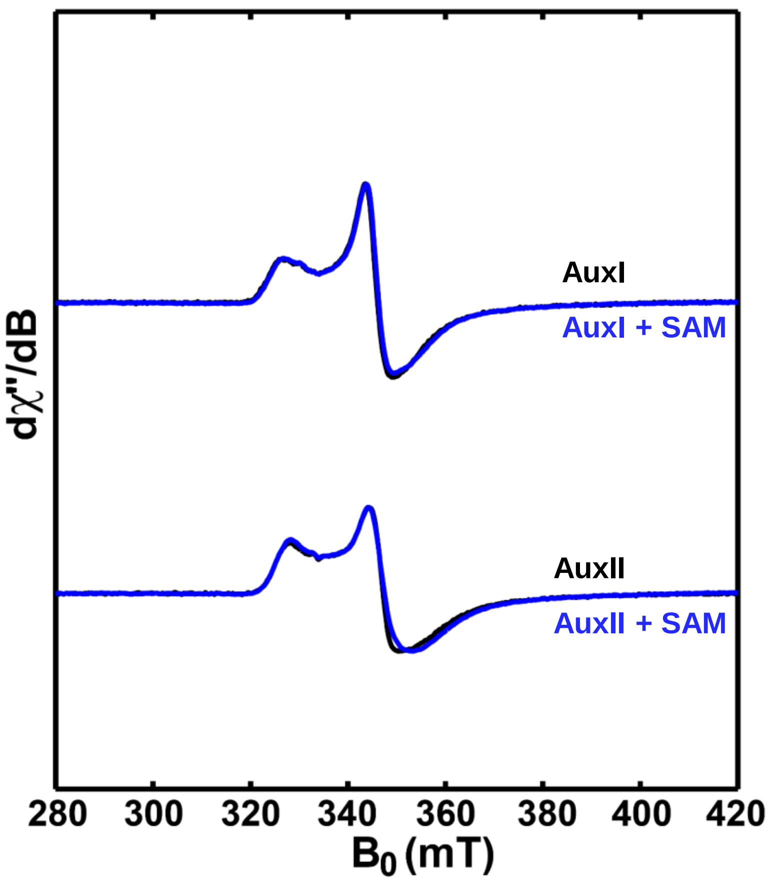

Supplementary Fig. 5 | X-band CW EPR spectra of DTH-reduced Gms mutant (black traces) that contains either AuxI cluster (C438AC123AC127A) or AuxII cluster (C123AC127AC370AC372A). The blue spectra correspond to the sample with the addition of SAM. Experimental parameters: temperature = 10 K; microwave frequency = 9.35 GHz; microwave power = 0.02 mW; conversion time = 40 ms; modulation amplitude = 0.05 mT; modulation frequency = 100 kHz.

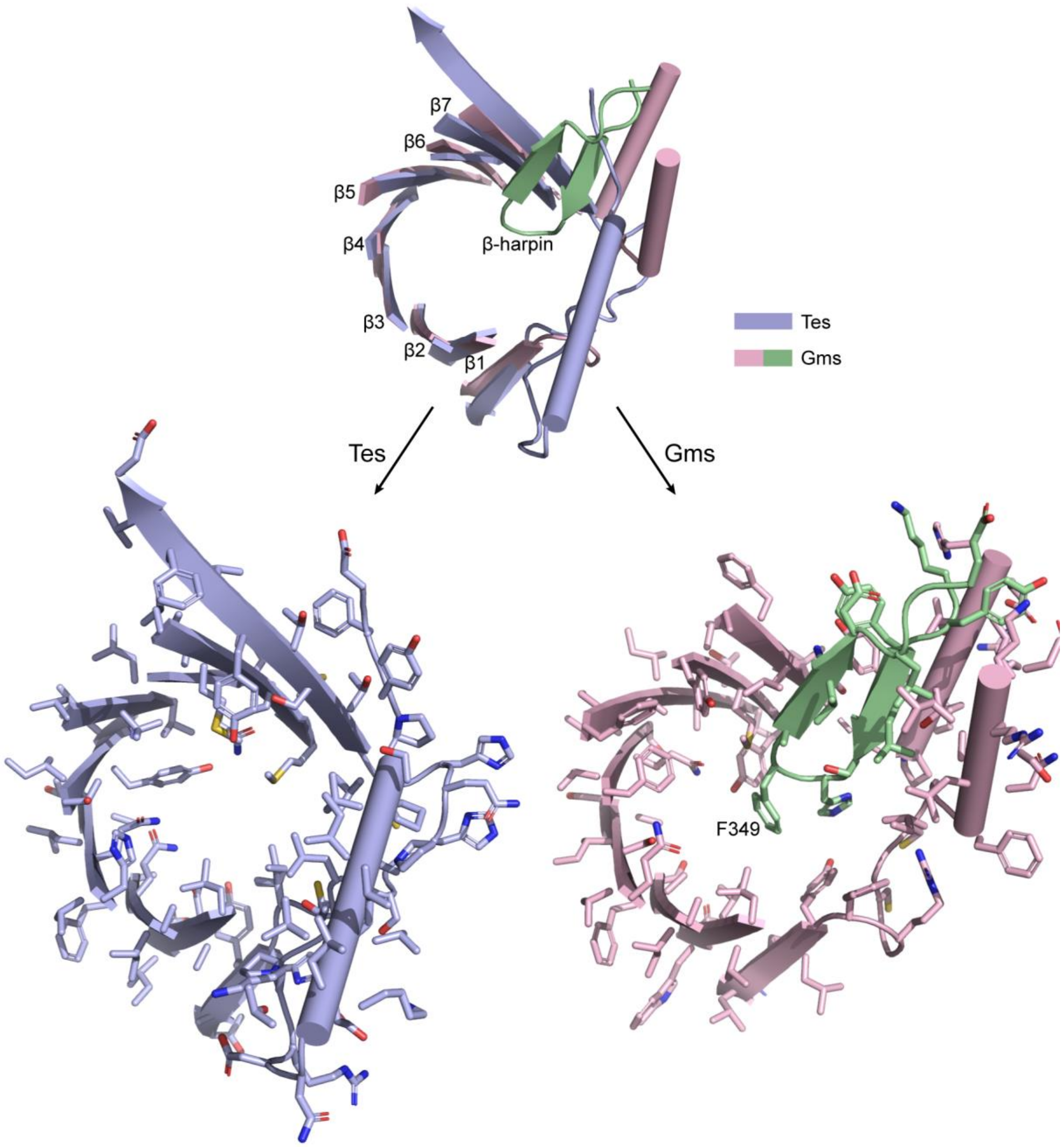

Supplementary Fig. 6 | The insertion of β-harpin results in a smaller binding pocket in Gms. The pockets of Tes (light blue) and Gms (light pink) are both composed of partial TIM barrel with hydrophobic residues. The β-hairpin Gms is colored in green.

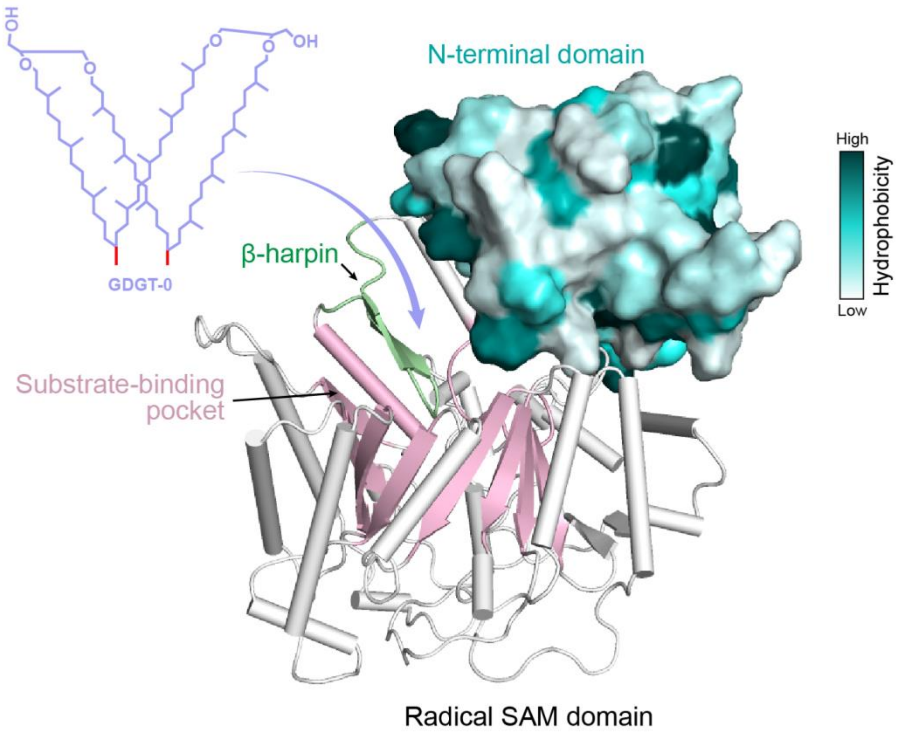

Supplementary Fig. 7 | Binding model of GMGT-0 to Gms. Through a V-shape folding, GMGT-0 inserts the two C atoms (in red) into the active site. In this process, the N-terminus of Gms needs to open up, and the hairpin needs to flip out, thereby providing sufficient substrate binding space for stabilizing the binding of GMGT-0 through hydrophobic interactions. Substrate-binding pocket and β-hairpin are in pink and green, respectively. The N-terminal of Gms is presented as surface and colored in teal according to the hydrophobicity of the residues.

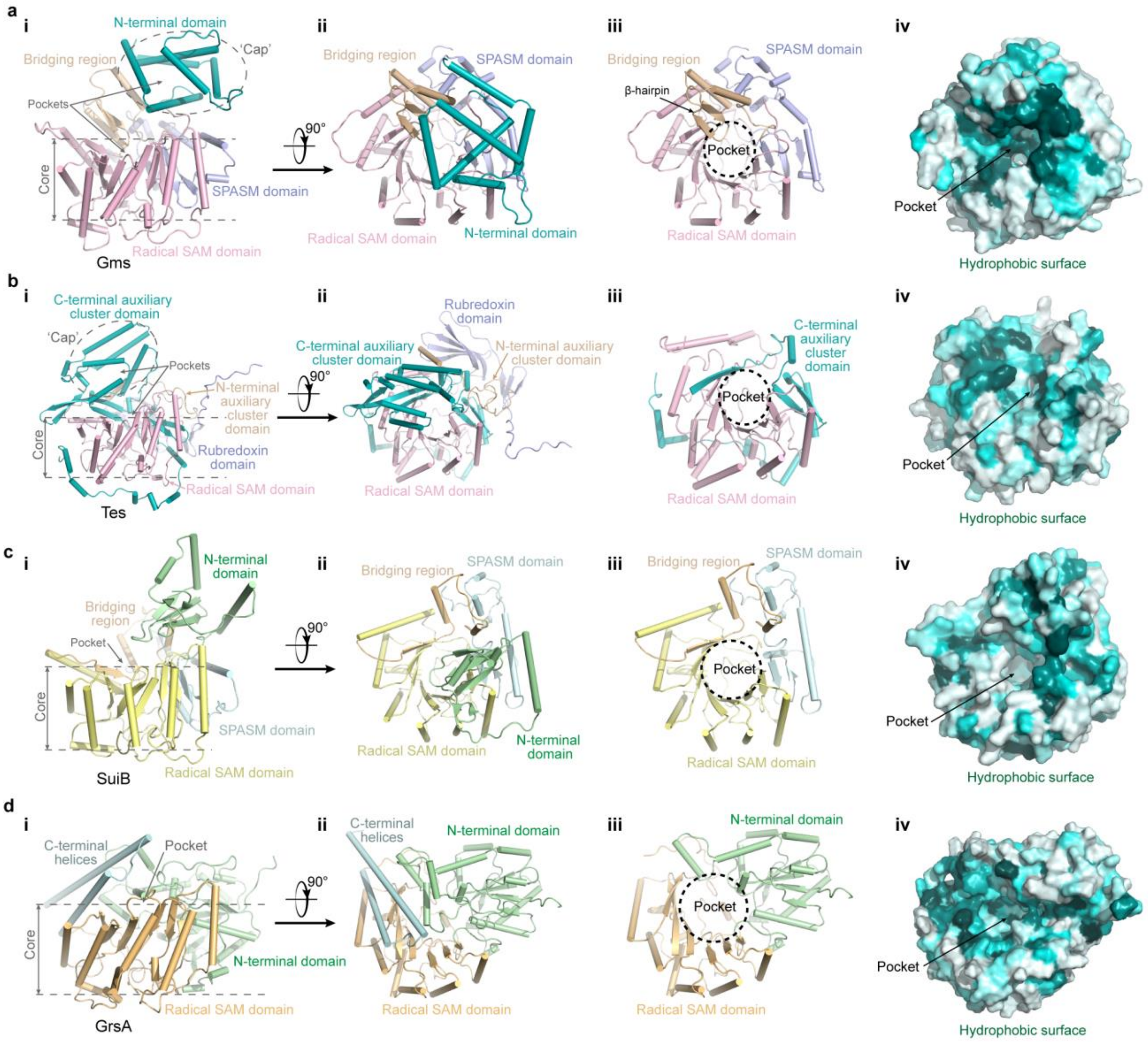

Supplementary Fig. 8 | Structural comparison of Gms, Tes, SuiB, and GrsA. Structures of Gms (**a**), Tes (**b**), SuiB (**c**), and GrsA (**d**) were aligned based on the radical SAM domain. Represent views for the overall structures are shown in panels i and ii. The substrate pockets in the radical SAM domain are shown as cartoon and hydrophobic surfaces in panels iii and iv.

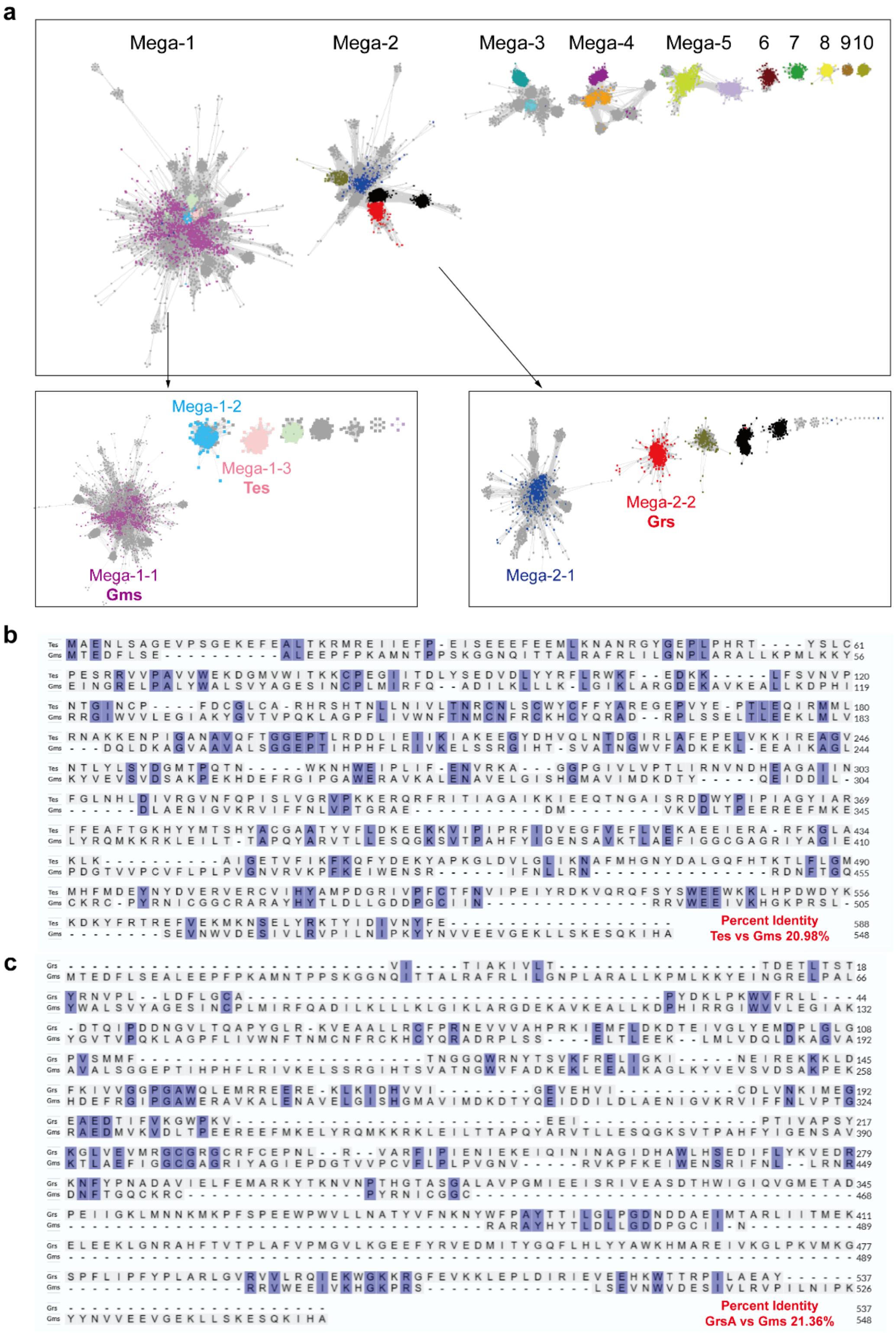

Supplementary Fig. 9 | The homology analysis of Gms, Tes, and GrsA sequences. (**a**) Subgroups identification of Gms, Tes and GrsA with RadicalSAM.org tool[1]. (**b**,**c**) Clustal Omega[2] was used to perform sequence alignment, and the Gms, GrsA and Tes protein sequences are from *Pyrococcus furiosus* DSM 3638 genome.

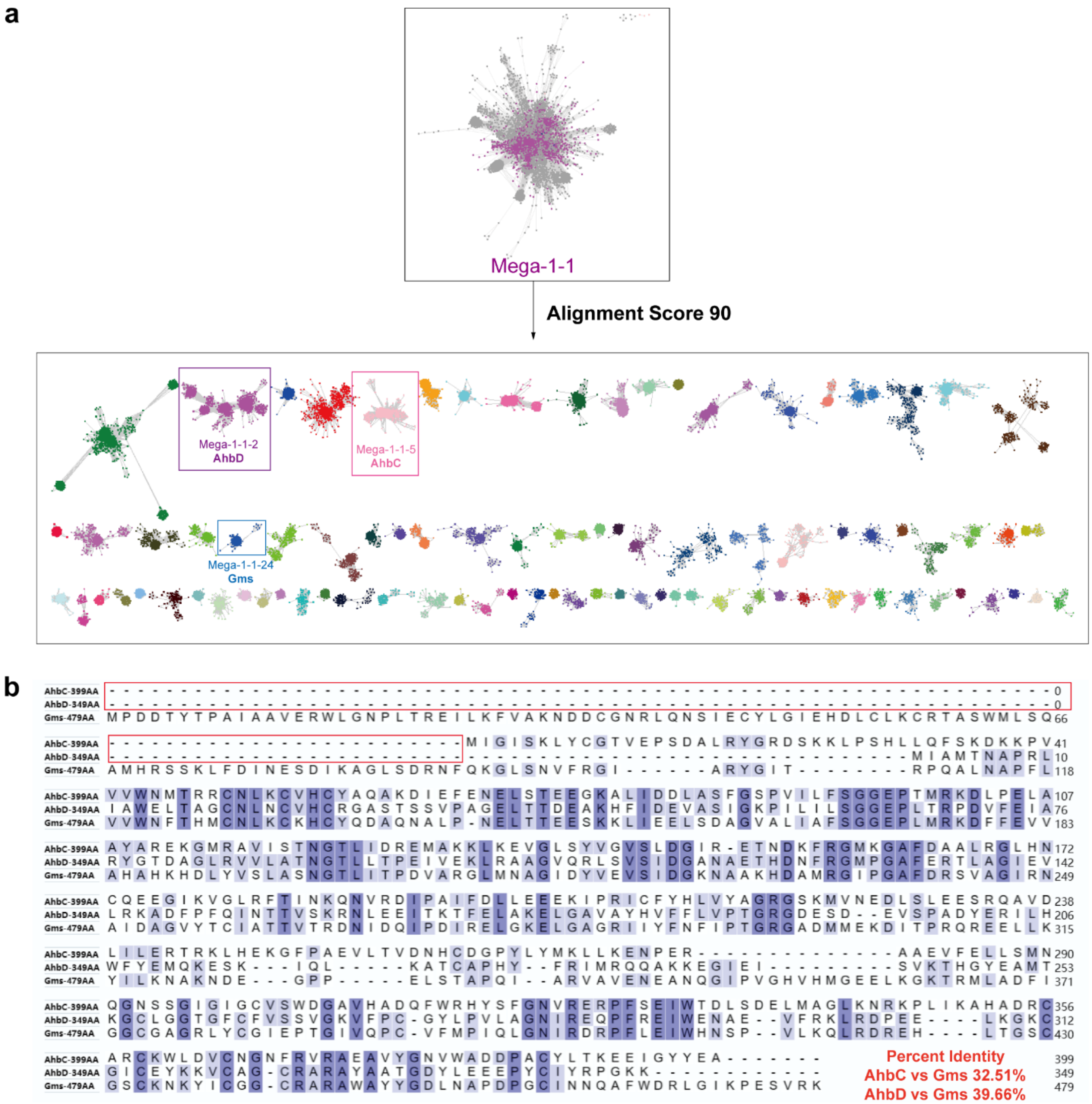

Supplementary Fig. 10 | The homology analysis of Gms and AhbC/AhbD. (**a**) Analysis with RadicalSAM.org tool[1], using the setting of alignment score 90 and segregating Gms form AhbC and AhbD within mega-1-1 subgroup. (**b**) Clustal Omega[2] was used to performed sequence alignment, and the Gms, AhbC, and AhbD protein sequences are from *Methanosarcina barkeri* genome**.**

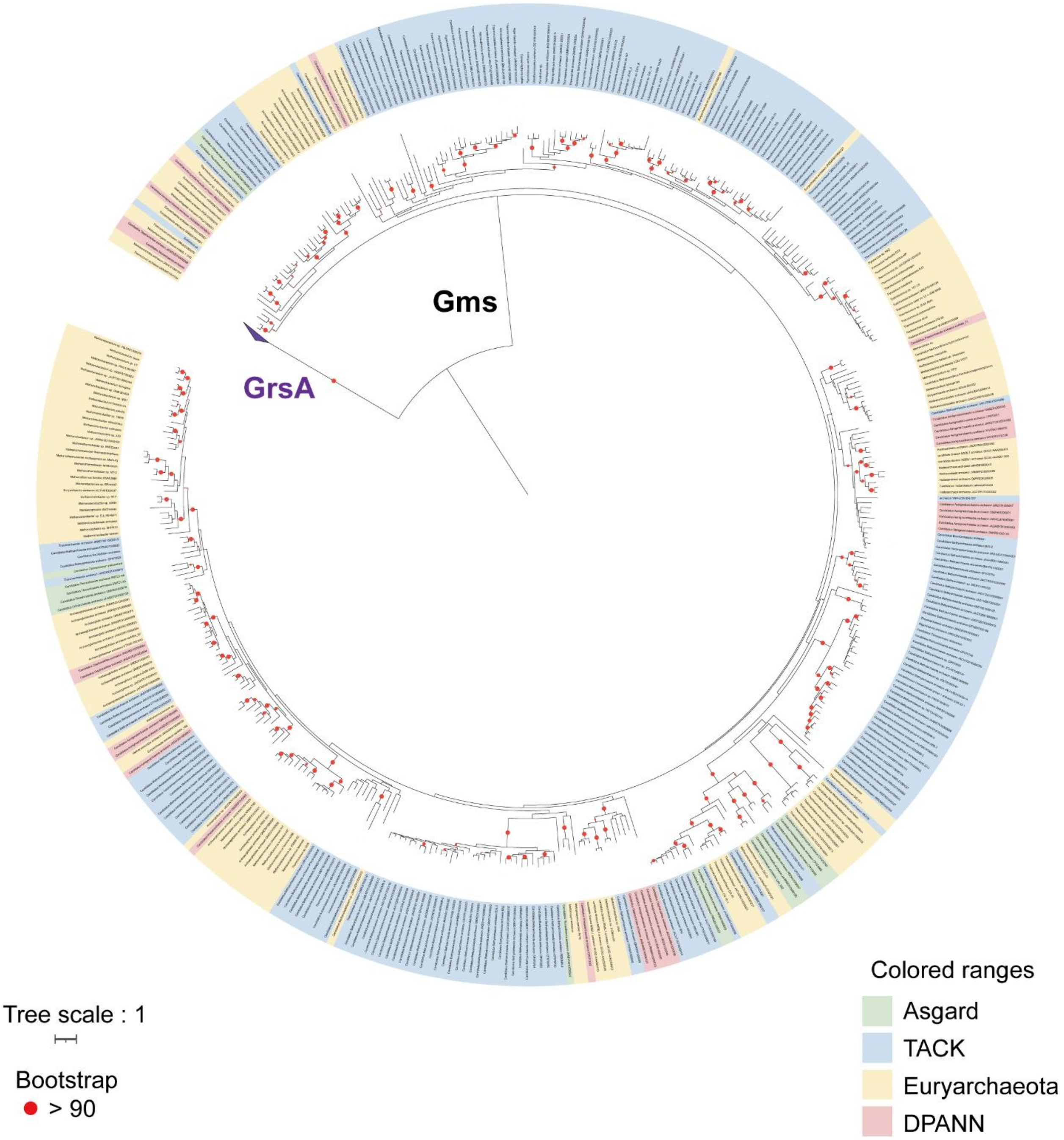

Supplementary Fig. 11 | Phylogeny of Gms proteins in Archaea domain. The maximum likelihood tree was generated by 1000 bootstrappable iterations using RAxML 8.2.12[3] software, based on 413 archaeal Gms homologs obtained by screening from the NCBI database. The 20 archaeal GrsA protein sequences were used as outgroup. Archaea superphyla include Asgard, TACK, Euryarchaeota and DPANN, which are color-coded in the tree. Red circles represent branches with a bootstrap value of > 90.

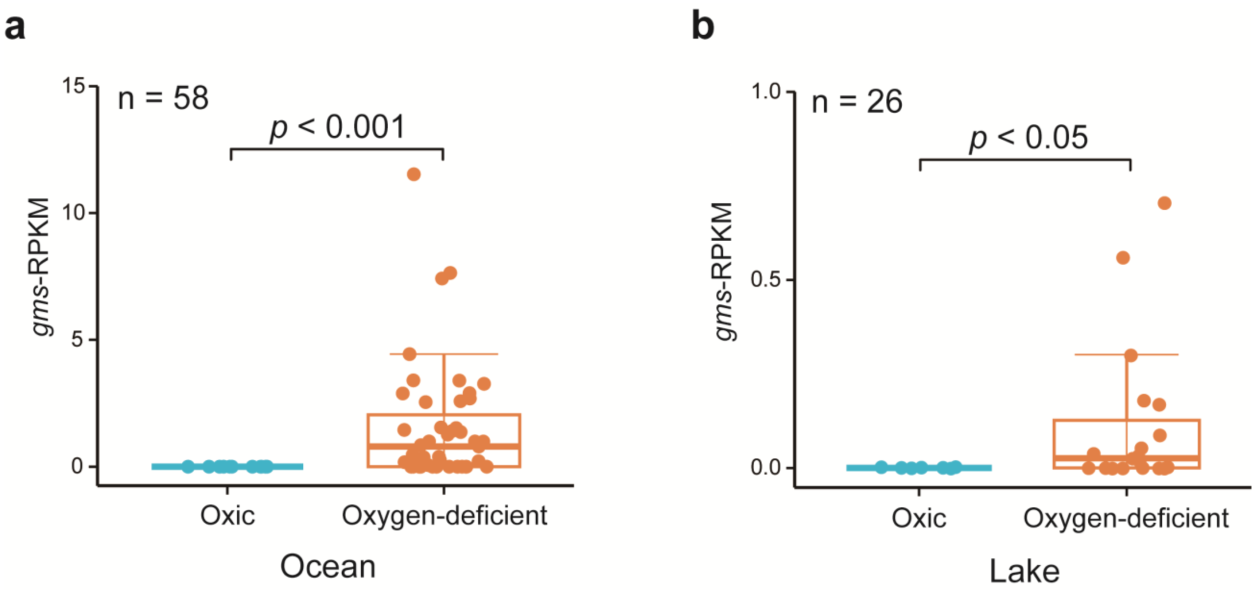

Supplementary Fig. 12 | The relative abundance of *gms*-RPKM in Oxic and Oxygen-deficient regions of ocean and lake. T-test was used to compare the oxic and oxygen-deficient ($p < 0.001$, n = 58) of ocean (**a**), the oxic and oxygen-deficient ($p < 0.05$, n = 26) of lake (**b**). The box's top and bottom delineate the third and first quartiles, respectively. The whisker stretches to a distance that is 1.5 times the Interquartile Range, starting from the box's edges.

Supplementary Table 1 | Radical SAM Proteins Comparison between GMGTs producing strains and non GMGTs producing strains.

GMGTs Production		NO GMGTs Production	
Strains	Protein ID	Strains	Protein ID
Pyrococcus furiosus DSM 3638	AAL80165.1	*Pyrococcus yayanosii* CH1	YP_004623180.1
	AAL80214.1		YP_004623187.1
	AAL80268.1		YP_004623324.1
	AAL80311.1		YP_004623492.1
	AAL80334.1		YP_004623538.1
	AAL80426.1		YP_004623571.1
	AAL80468.1		YP_004623695.1
	AAL80755.1		YP_004623702.1
	AAL80771.1*		YP_004623788.1
	AAL80859.1		YP_004623809.1
	AAL80967.1		YP_004623902.1
	AAL80974.1		YP_004624001.1
	AAL81049.1		YP_004624107.1
	AAL81173.1		YP_004624113.1
	AAL81282.1		YP_004624153.1
	AAL81401.1		YP_004624227.1
	AAL81513.1		YP_004624341.1
	AAL81521.1		YP_004624416.1
	AAL81578.1		YP_004624520.1
	AAL81594.1		YP_004624563.1
	AAL81752.1		YP_004624619.1
	AAL81842.1		YP_004624679.1
	AAL81847.1		
	AAL82025.1		
	AAL82036.1		
	AAL82096.1		
	AAL82157.1		
	AAL82184.1		
	AAL82188.1		
Thermococcus guaymasensis DSM 11113	WP_062371178.1	*Thermococcus kodakarensis* KOD1	YP_182580.1
	WP_062371123.1		YP_182601.1
	WP_062370681.1		YP_182703.1
	WP_062370497.1		YP_183088.1
	WP_062370462.1		YP_183116.1
	WP_062369928.1		YP_183266.1
	WP_062369926.1*		YP_183306.1
	WP_062369925.1		YP_183310.1
	WP_062373931.1		YP_183477.1
	WP_062373843.1		YP_183548.1
	WP_062373718.1		YP_183611.1
	WP_062373231.1		YP_183641.1

	WP_062373044.1		YP_183825.1
	WP_062372388.1		YP_183987.1
	WP_062372270.1		YP_184084.1
	WP_062372197.1		YP_184179.1
	WP_062372081.1		YP_184220.1
	WP_062371952.1		YP_184477.1
	WP_062371856.1		YP_184522.1
	WP_062371570.1		YP_184558.1
	WP_062371406.1		YP_184573.1
	WP_062371397.1		YP_184638.1
			YP_184661.1
			YP_184709.1
			YP_184712.1

* The protein IDs highlighted in red are Gms candidate proteins.

Supplementary Table 2 | Strains used in this study.

Strains		Genotype	Source or reference
Methanococcus maripaludis	S001	Expression host containing ORF1 from pURB500 integrated into the *M. maripaludis* S2 genome	4
	HH004	S001+pMEV4-*maeo_0574*	5
	YN001	S001+pMEV4-*METOK_RS04425- maeo_0574*	This work
Escherichia coli	BL21(DE3) *Δ iscR*	*ΔiscR*	6
	DH10B	F^{-} *endA1 recA1 galE15 galK16 nup GrpsL ΔlacX74 Φ80lacZΔM15 araD139 Δ(ara,leu)7697 mcrA Δ(mrr-hsdRMS mcrBC)λ*$^{-}$	Paula V. Welander (Stanford University)
S. acidocaldarius		*S. acidocaldarius ΔgrsAΔgrsB*	7

Supplementary Table 3 | Plasmids used in this study.

Plasmids	Description	Source or reference
pMEV4-*maeo_0574*	*M. maripaludis* expression plasmid containing *maeo_0574* gene.	5
pMEV4-*METOK_RS04425-maeo_0574*	HH004 expression plasmid containing *METOK_RS04425* gene, which was amplified by PCR with primers P1F/R and cloned into the AfeI site of pMEV4-*maeo_0574*.	This work
pET-21b (+)-*METOK_RS04425*	pET-21b (+) expression plasmid containing *METOK_RS04425* gene.	This work
pET-21b (+)-*METOK_RS04425-ΔRS*	pET-21b (+)-*METOK_RS04425* expression plasmid which was mutant C123A C127A by PCR with primers *ΔRS*-F/1R and *ΔRS*-R/1F.	This work
pET-21b (+)-*METOK_RS04425-ΔAuxI*	pET-21b (+)-*METOK_RS04425* expression plasmid which was mutant C370A C372A by PCR with primers *ΔAuxI*-F/1R and *ΔAuxI*-R/1F.	This work
pET-21b (+)-*METOK_RS04425-ΔRS-ΔAuxI*	pET-21b (+)-*METOK_RS04425-ΔRS* expression plasmid which was mutant C370A C372A by PCR with primers *ΔAuxI*-F/1R and *ΔAuxI*-R/1F.	This work
pET-21b (+)-*METOK_RS04425-ΔRS-ΔAuxII*	pET-21b (+)-*METOK_RS04425-ΔRS* expression plasmid which was mutant C438A by PCR with primers *ΔAuxII*-F/1R and *ΔAuxII*-R/1F.	This work
pET-21b (+)-*METOK_RS04425-ΔAuxI-ΔAuxII*	pET-21b (+)-*METOK_RS04425-ΔAuxI* expression plasmid which was mutant C438A by PCR with primers *ΔAuxII*-F/1R and *ΔAuxII*-R/1F.	This work

Supplementary Table 4 | Primers used in this study.

Primers	Sequence (5' to 3')
ΔRS-F	GATGTGACCTATGCGGCGAACCTGCGCGCGAAACATTGCTATGCG
ΔRS-R	GTTTCGCGCGCAGGTTCGCCGCATAGGTCACATC
ΔAuxI-F	AACTTTATTGGCGGCGCCGGCGCCGGCCGCTTTTATCTG
ΔAuxI-R	GCGGCCGGCGCCGGCGCCGCCAATAAAGTT
ΔAuxII-F	ATGTATAAATATGTGGCCGGCGGCTGCCGCGCGCGCGCGA
ΔAuxII-R	GCGCGGCAGCCGCCGGCCACATATTTATACAT
1F	GAGTTGCATGATAAAGAAGACAGTCATAAG
1R	CAACGCAATTAATGTAAGTTAGCTCACTCATTAG
P1F	GGCTTATGAAATTTGTTAAAATTTAGC
P1R	TACCCAAATATTTCAATGAATATTTAG

Supplementary References

1 Oberg, Nils. et al. RadicalSAM. org: a resource to interpret sequence-function space and discover new radical SAM enzyme chemistry. *ACS Bio Med Chem Au* **2**, 22-35 (2021).

2 Sievers, Fabian, et al. Fast, scalable generation of high-quality protein multiple sequence alignments using Clustal Omega. *Mol. Syst. Biol.* **7**, 539 (2011).

3. Stamatakis, A. RAxML version 8: a tool for phylogenetic analysis and post-analysis of large phylogenies. *Bioinformatics* **30**, 1312-1313 (2014).

4 Tumbula, D. L., Makula, R. A. & Whitman, W. B. Transformation of *Methanococcus maripaludis* and identification of a Pst I-like restriction system. *FEMS Microbiol. Lett.* **121**, 309-314 (1994).

5 Zeng, Z. et al. Identification of a protein responsible for the synthesis of archaeal membrane-spanning GDGT lipids. *Nat. Commun.* **13**, 1545 (2022).

6 Tao, L. et al. Radical SAM enzyme HydE generates adenosylated Fe (I) intermediates en route to the [FeFe]-hydrogenase catalytic H-cluster. *J. Am. Chem. Soc.* **142**, 10841-10848 (2020).

7 Zeng, Z. et al. GDGT cyclization proteins identify the dominant archaeal sources of tetraether lipids in the ocean. *Proc. Natl. Acad. Sci.* **116**, 22505-22511 (2019).
