# Supplementary material for: Biosynthesis of GMGT lipids by a radical SAM enzyme associated with anaerobic archaea and oxygen-deficient environments
Source: Nat Commun. 2024 Jun 19;15:5256. doi: 10.1038/s41467-024-49650-x (PMC11186832; doi:10.1038/s41467-024-49650-x)
Supplement: Supplementary file 3 — Description of Additional Supplementary Files [file 41467_2024_49650_MOESM3_ESM.pdf]

Description of Additional Supplementary Files:

Supplementary Dataset 1: AlphaFold2 model of Gms protein

Supplementary Dataset 2: AlphaFold2 model of Tes protein

Supplementary Dataset 3: AlphaFold2 model of GrsA protein
